# Microbial bioformulation: a microbial assisted biostimulating fertilization technique for sustainable agriculture

**DOI:** 10.3389/fpls.2023.1270039

**Published:** 2023-12-12

**Authors:** Amir Khan, Ajay Veer Singh, Shiv Shanker Gautam, Aparna Agarwal, Arjita Punetha, Viabhav Kumar Upadhayay, Bharti Kukreti, Vindhya Bundela, Arun Kumar Jugran, Reeta Goel

**Affiliations:** ^1^ Biofortification Lab, Department of Microbiology, College of Basic Sciences and Humanities, Govind Ballabh Pant University of Agriculture and Technology, U.S. Nagar, Uttarakhand, India; ^2^ School of Environmental Science and Natural Resource, Dehradun, Uttarakhand, India; ^3^ Department of Microbiology, College of Basic Sciences and Humanities, Dr. Rajendra Prasad Central Agriculture University, Samastipur, India; ^4^ G. B. Pant National Institute of Himalayan Environment (GBPNIHE), Garhwal Regional Centre, Srinager, Uttarakhand, India; ^5^ Department of Biotechnology, Institute of Applied Sciences and Humanities, GLA University, Mathura, Uttar Pradesh, India

**Keywords:** bioformulation, plant growth promoting microbes, carrier, viability, sustainability

## Abstract

Addressing the pressing issues of increased food demand, declining crop productivity under varying agroclimatic conditions, and the deteriorating soil health resulting from the overuse of agricultural chemicals, requires innovative and effective strategies for the present era. Microbial bioformulation technology is a revolutionary, and eco-friendly alternative to agrochemicals that paves the way for sustainable agriculture. This technology harnesses the power of potential microbial strains and their cell-free filtrate possessing specific properties, such as phosphorus, potassium, and zinc solubilization, nitrogen fixation, siderophore production, and pathogen protection. The application of microbial bioformulations offers several remarkable advantages, including its sustainable nature, plant probiotic properties, and long-term viability, positioning it as a promising technology for the future of agriculture. To maintain the survival and viability of microbial strains, diverse carrier materials are employed to provide essential nourishment and support. Various carrier materials with their unique pros and cons are available, and choosing the most appropriate one is a key consideration, as it substantially extends the shelf life of microbial cells and maintains the overall quality of the bioinoculants. An exemplary modern bioformulation technology involves immobilizing microbial cells and utilizing cell-free filters to preserve the efficacy of bioinoculants, showcasing cutting-edge progress in this field. Moreover, the effective delivery of bioformulations in agricultural fields is another critical aspect to improve their overall efficiency. Proper and suitable application of microbial formulations is essential to boost soil fertility, preserve the soil’s microbial ecology, enhance soil nutrition, and support crop physiological and biochemical processes, leading to increased yields in a sustainable manner while reducing reliance on expensive and toxic agrochemicals. This manuscript centers on exploring microbial bioformulations and their carrier materials, providing insights into the selection criteria, the development process of bioformulations, precautions, and best practices for various agricultural lands. The potential of bioformulations in promoting plant growth and defense against pathogens and diseases, while addressing biosafety concerns, is also a focal point of this study.

## 1 Introduction

In the last few decades, rampant chemical fertilization and biomagnification of hazardous chemicals in the food chain has posed a threat to human health and destroyed the health of the soil. The deterioration of soil fertility and decline in the indigenous beneficial soil microbial population led to decreased crop production. Hence, an alternative and green approach is needed to maintain agricultural productivity without reliance on chemical fertilization. The use of microbial bio-formulations offers an alternative approach for utilizing beneficial plant microorganisms to achieve good plant growth and productivity. The use of bio-formulated products, especially biofertilizers, has been widely popularized as an alternative to the agrochemicals (Khan et al., 2020a; Pathak et al., 2022; Ayilara et al., 2023). Therefore, the term bio-formulation can be represented as the ‘development of material containing living but valuable microbial strains, using suitable carrier materials for their productive use in agriculture, industry, bioremediation, etc (Balla et al., 2022). The key ingredients of a bio-formulated product/bioformulation are potential microbes, possessing plant growth promoting properties including nutrient solubilizers, nitrogen fixers, biocontrol agents, and bioremediation (Pirttila et al., 2021). The major goals of microbial formulations preparation are: (i) to create an appropriate environment for the bioinoculants functioning, ii) to provide physical and chemical protection for an extended period of time to circumvent a rapid reduction in cell viability during storage, (ii) to support the competition of inoculants with the indigenous soil microbiota, and (iii) to reduce losses engendered from depredation by the local micro-fauna. Another goal, however, is to provide a sufficient source of live bioinoculant cells that are accessible for interaction with plants and the soil microbiome (Vassilev et al., 2020). It has been observed that direct use of plant beneficial microorganisms in the green house or small scale is fine but on field or large scale, viability issue of the microorganisms gets enhanced. Indeed, it is necessary to obtain a significant number of microbial cells (at least 10^6^-10^7^) in order to obtain a positive response of the formulated product (Bashan et al., 2014; Vassilev et al., 2020). The abiotic substrates, which have the ability to provide a safer environment for microbial cells and can accommodate viable and physiologically active cells, are called as carrier substances. Solid or liquid materials are used as ‘carriers’ for the development of various microbial formulations, depending on the product type (Naik et al., 2020). The solid formulations are produced in solid, powdery, or granular form and are based on either inorganic or organic carriers. Various carrier materials such as peat, vermiculite, coal, compost, perlite, agro-industrial waste, polysaccharides, etc. are used to produce the most important solid formulations. In contrast, liquid-based formulations also contain microbial cultures with desirable properties, modified with additives that improve the viscosity, constancy, and dispersibility of the cell suspension (Mishra and Arora, 2016). In recent years, formulation technologies have paid more attention to the immobilization of cells, since the tactic of gel-cell immobilization is the technological solution that can better ensure the quality and standardization of the formulated product. In addition, particular attention has recently been paid to cell-free formulations (Tewari et al., 2020). These formulations resemble fermentation broth and encompass various metabolic products, including metal chelators (siderophores), antibiotics, enzymes, notably those with lytic capabilities, toxins, and soluble phosphate. Collectively, these components have the potential to exert a beneficial influence on plant growth. Delivery of bioformulations is a mandatory step, done either by inoculating the soil directly or by treating plants/seeds (Rocha et al., 2019a). The escalating concern over the inadequate uptake of chemical fertilizers by plants and their detrimental impact on ecosystems, alongside a global rise in apprehension regarding pollution, greenhouse gas accumulation, and an increased emphasis on plant-based food production, has led to a surging demand for biofertilizer agents. Farmers are increasingly embracing biofertilizers to sustainably and organically cultivate their crops. To date, numerous biofertilizers have been successfully commercialized for various environmental conditions and crops. However, a significant obstacle to the widespread success of biofertilizers in agroecosystems is the lack of knowledge in selecting and correctly applying them. This knowledge gap erodes the confidence of farmers in biofertilizers. Hence, there is a critical need to disseminate knowledge within farming communities about the scientifically sound methods of selecting and applying correct microbial bioformulations according to their native environment and crops.

## 2 Stages of bio-formulation preparation

**Figure 1:**
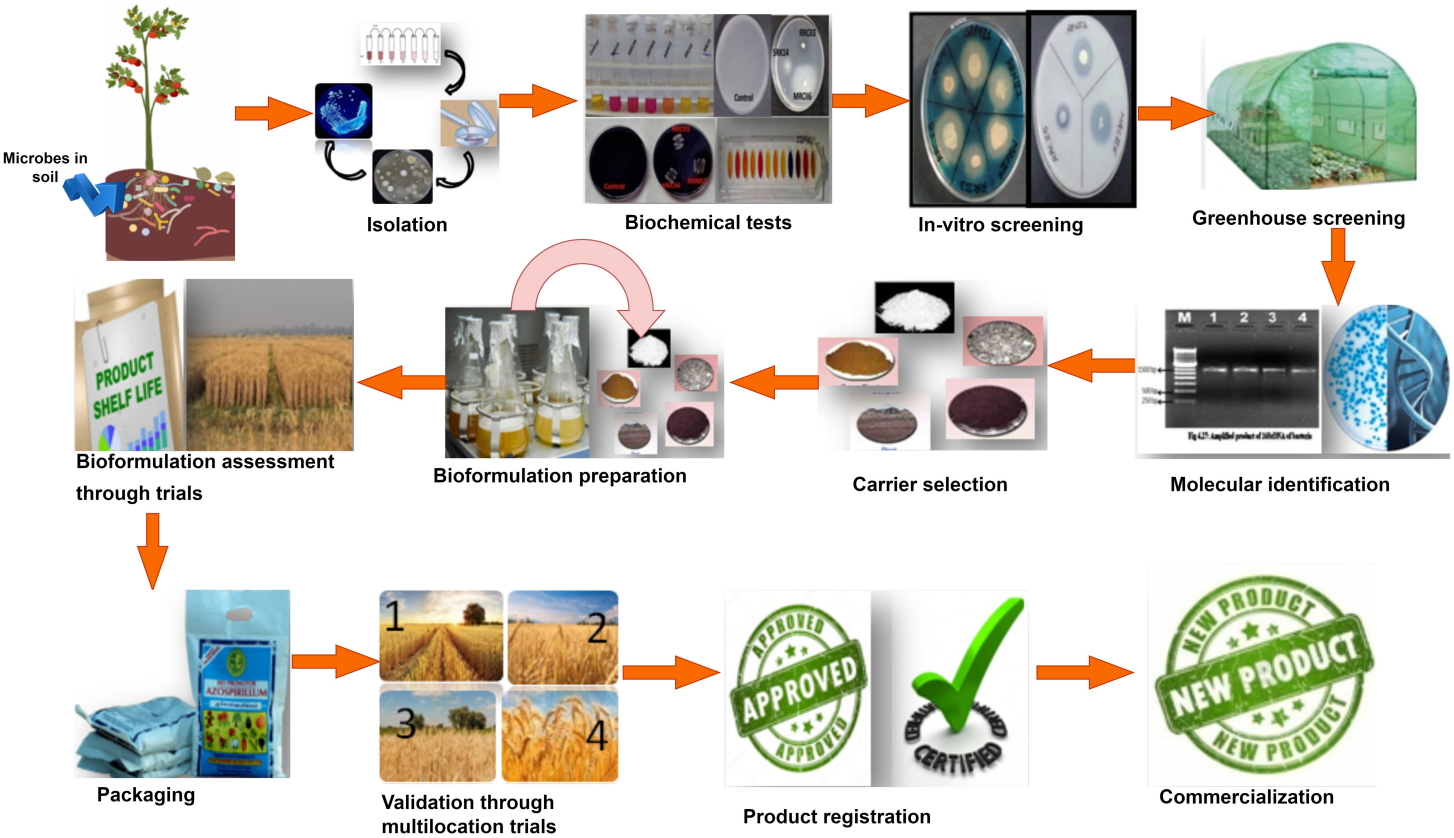
Diagrammatic representation of steps involved in bioformulation development.

Bioformulation’s performance greatly depends on multiple dynamics under field conditions, including microbial composition, the carrier used for bioformulation preparation, delivery method, application strategies, and sustenance of microbial strains in native soil and plant ecosystem, which are being selected during the development of bioformulations (Bargaz et al., 2018). The development of effective and efficient bioformulation mainly depends on the constituents used to prepare the bioformulation, which comprises potential beneficial microbial strains, a suitable carrier, and an adjuvant (Aamir et al., 2020). Steps considered for the bioformulation development are summarized below (**Figure 1**):

### 2.1 Selection of potential microbial candidate

The selection of microorganisms for bioformulation development is an essential step for the startup process of bioformulation. Primary selection of microbial strains occurs on the grounds of their plant growth promoting (PGP), antagonistic, degradation potential, and any other useful properties (Wong et al., 2019; Singh et al., 2020). In order to select potential candidates, various microbial sources such as soil, water, and any other specific substances are being used for isolation, they were tested for various properties such as siderophore and lytic enzymes production, nutrient solubilization, production of phytohormones and antibiotics, xenobiotics degradation or heavy metal bioremediation, etc. (Vassilev et al., 2015) which make them a potential candidate for bioformulation development. Further, these microbes were tested for *In vitro* and *In vivo* plant growth promotion and bioremediation properties assessment. In addition, some desirable characteristics must be met with the microbial strains, including genetic stability, physiological adaptability with the host environment, extended self-life, survival capability under harsh conditions, efficient colonization with the host plant, non- pathogenicity, etc (Chakraborty, 2020). Sometimes, instead of a single microbe, more than one microbial strain is used for bioformulation preparation. Species of diverse genera i.e. *Acetobacter*, *Arthrobacter*, *Azotobacter*, *Azospirillum*, *Bacillus*, *Burkholderia*, *Clostridium*, *Enterobacter*, *Flavobacterium*, *Frankia*, *Hydrogenophaga*, *Kluyvera*, *Microcoleus*, *Phyllobacterium*, *Pseudomonas*, *Serratia*, *Streptomyces*, *Rhizobium, Trichoderma*, etc. have been already reported for splendid PGP potential and being considered for bioformulation preparation (Soni et al., 2017; Alawiye and Babalola, 2019; Suyal et al., 2019; Jeyakumar et al., 2020; Comite et al., 2021). Further, many microorganisms such as *Penicillium bilaiae*, *Rhizobium leguminosarum*, *Bradirhizobium japonicum, Bacillus amyloliquefaciens, Trichoderma virens* having multiple plant growth and biocontrol properties have been commercialized as biofertilizers through various organizations.

### 2.2 Carrier selection and bioformulation assemblage

A suitable carrier is an important constitute of bioformulation preparation. It acts as delivery material for live microbial strains during the processing from laboratory to field. Individually or compositely, suitable inorganic/organic or synthetic carriers viz. peat, coal, clays, talc, vermiculite, charcoal, cellulose, sawdust, wheat bran, alginate beads, rice husk, polyacrylamide gel, calcium sulfate, silica gel etc. can be used to support microbial growth and effective delivery of desired microbes into the field (Vishwakarma et al., 2018; **Table 1**). Different carrier materials demonstrate multiple effects on microbial viability and the efficacy of delivery. Carriers used for bioformulation preparation can significantly impact the shelf life, bioavailability, release rate, and overall performance of the bioinoculants-based bio-formulation. Therefore, compatibility with the microorganisms, potential toxicity or immune response from the carrier should be prioritized during the selection of the carrier material. The carrier should also protect bioactive compounds from deterioration brought on by external elements including heat, light, and moisture. The carrier should be able to permit controlled release of the bioactive chemicals depending on the intended application in order to increase their bioavailability. Additionally, the carrier material’s particle size merits attention because smaller particles typically give faster dispersibility and dissolving rates. It’s critical to assess if the chosen carrier can be processed and scaled up effectively while remaining ecologically friendly in the context of commercial manufacturing (Singh et al., 2020; Aloo et al., 2022; Rojas-Sánchez et al., 2022);. During bioformulation preparation, the sterilization of carriers is an essential step. For this, gamma irradiation at a dose rate of 4.0 kGy for 1 h or autoclaving at 121°C for 20-30 min, is the most suitable way of carriers sterilization, and being used for selected carriers viz. rice husk, wheat bran, clay, peat moss and the mixture of peat moss and vermiculite (El-Fattah et al., 2013; Sahai et al., 2019). The sterilized carrier is mixed with actively grown microbial strains and air dried overnight to retain 15-20% moisture content, which is essential to lower down the microbial metabolic activities (Samavat et al., 2014). Furthermore, the mixture is packed and sealed in pre-sterilized i.e. autoclaving or gamma irradiation, polypropylene bags and stored at room temperature with 80% relative humidity (Arora et al., 2010; Suryadi et al., 2013; Namsena et al., 2016; Bargaz et al., 2018). A good bioformulation must contain at least ~ 10^7^ cfu/g of microbial cells (Jambhulkar and Sharma, 2014; Samavat et al., 2014).

**Table 1:** Examples of carrier-based microbial bio-formulation tested on various crops with their advantageous effects.

Carrier and Additive	Microbial Inoculant	Crop	Effect	Reference
Talc + CMC	*Bacillus* sp.*; P. putida, P. jesinni* MP1	Cowpea, lady’s finger, Cucumber, lettuce, Chickpea	Enhance seed germination and plant growth, Stabilize microbial survival, Increase soil nutrient status	Basheer et al. (2019); Joshi et al. (2019)
Talc	*P. fluorescens*	Rice	Enhance plant growth and nutrient status, reducing disease index in rice	Saravanakumar et al. (2007)
Talc + chitin	*P. fluorescens*	Mungbean	Increase plant growth	Saravanakumar et al. (2007)
Talc + Xanthum Gum	*Paenibacillus alvei*	Cotton	Enhance plant growth, reducing the disease caused by *Thielaviopsis basicola*	Schoina et al. (2011)
Saw-dust+ CMC	*Ensifer meliloti*, *Bradyrhizobium* sp.	*Mucuna pruriens*	Nodulation enhancement, Increased survival	Aeron et al. (2012)
Industrial Oxalic Acid	*B. japonicum*	Soybean	Enhanced plant growth and nodulation, Increase in shelf life	Rebah et al. (2007)
Perlite + Arabic gum	*R. leguminosarum, B. megaterium*	Soybean	Increase survival at low temp	Daza et al. (2000)
Canola oil as emulsion	*Sinorhizobium meliloti*	Alfalfa	Enhance survival, Increased nodulation	John et al. (2011)
Alginate + humic acid	*P. putida, B. subtilis*	*Lectuca sativa*	Enhance plant growth	Rekha et al. (2007)
Peat +Vermiculite	PGPB (six consortia)	Melons	Enhance plant growth, Provide disease tolerance to plants	Kokalis-Burelle et al. (2003)
Peat + Chitin or A. niger mycelium	*B. subtilis, Klebsiella pneumoniae*	Groundnuts, Pigeon pea	Increase seed germination, high multiplication, provide efficiency against disease	Manjula and Podile (2001)
Peat + Sugar	*A. brasilense*	Wheat	Enhance in plant growth	Piccinin et al. (2013)
Clay soil + CMC/Gum arabic	*Bradyrhizobium japonicum, B. megaterium*	Soybean	Enhancement in plant growth, Increase in survival of microbes	Albareda et al. (2008)
Clay soil + elemental S	Thiobacillus and *Rhizobium* sp.	Ground nut	Enhancement in plant growth and nodulation	Anandham et al. (2007)
CMC/corn starch + MgO	*A. amazonense*, *G. diazotrophicus*, *H. seropedicae*, *H. rubrisubalbicans*, and *B. tropica*	Sugarcane	Increase in shelf life of bacteria, Increase colonization	Da Silva et al. (2012)

### 2.3 Desiccation tolerance testing

Bacterial desiccation is a natural abiotic stress condition, usually occurring in the environment by freezing, heating, or drying and rewetting conditions of soil due to low precipitation or irrigation. Hence the bioformulation must show sustenance against this for efficient effect. Several spore-forming microbes could survive under desiccation, but the condition becomes lethal, especially for non-spore forming bacteria. Although, multiple physiological mechanisms have been observed behind the desiccation tolerance in non-spore forming microbial strains, which include synthesis of a compatible solute such as disaccharide trehalose or hydroxyl pyrimidine hydroxyl ectoine (Roder et al., 2005; Narvaez-Reinaldo et al., 2010; Khan and Singh, 2021), production of heat-shock proteins, enzymes and exopolysaccharides modification or repair of DNA mechanisms (Berninger et al., 2018). Hence, desiccation tolerance has great biotechnological interest in microbial cellular stabilization which allows the long-term storage of formulated products for commercial uses. Microbial inherent desiccation tolerance could be improved during the bioformulation process by applying some strategies including drying methods i.e. freeze-drying, vacuum-drying, spray-drying, fluidized bed-drying, and air-drying, the addition of external protectants, triggering of stress adaptation, triggering of exopolysaccharide secretion, and indirect protection by “helper” microbial strains (Berninger et al., 2018). Drying method is well known efficient way for long term storage. By following suitable drying conditions, the quality and self-life of the products can be enhanced equally. Further, disaccharide such as trehalose is an example of such protectant. During desiccation, trehalose forms hydrogen bonds with other proteins in the absence of water, preventing protein denaturation (García, 2011). Moreover, the implication of sublethal stress, including the variation of pH, temperature, depletion of nutrients, anoxic conditions, and salt stress conditions has been suggested before the desiccation to activate the cellular protective mechanisms (Liu et al., 2014).

### 2.4 Storage stability testing

The determination of the storage ability of bioformulation is an essential and critical factor in bioformulation efficacy. Usually, the self-life of the product and its microbial stability can be expected from 6 to 12 months (Berninger et al., 2018). The additives and low temperature storage are essential factors for the survival and stability of bioformulation. The stability test is performed through serial dilution plating at different time intervals and colony forming unit (CFU) estimation is done, which should not be less than 10^4^ CFU per gram sample (Wong et al., 2019). Several encapsulation materials, a wide temperature range, and different environmental conditions are being used to test the bioformulation sustenance capacity, which determines the shelf life of bioformulation. Further, bioformulations packaged into suitable bags to carry efficient microbes and to maintain humidity.

### 2.5 Validation, registration and approval

After bioformulation preparation and its successful demonstration in fields, the bioformulation is further processed for validation, in which the same bioformulation is tested through multi-locational field trials. After validation, the bioformulation needs registration through patent and risk-related approval before commercialization.

## 3 Carriers and adjuvant used for bio-formulation

Carrier and adjuvant impart a major role in microbial survival during production, storage, and application processes (John et al., 2011; Herrmann and Lesueur, 2013).

### 3.1 Carrier

The success of any bioformulation mainly depends upon the carrier or bulking agent, which is the 2^nd^ most prominent component used in the preparation of bioformulation. The carrier material provides a protective environment and energy source for microbial growth and development and guarantees the successful release of the bacterial cells after the application. Carriers used in seed treatment should have good adhesion capacity with seeds to get better efficacy (Hegde and Brahmaprakash, 1992). The characteristics of the carrier include being cost-effective, easy to be processed, chemically stable, good moisture absorption and buffering capacity, non-toxic for both plant and microbes, and ensuring bacterial cell viability after a specified period of storage (minimum 2-3 months). There are varieties of carriers used nowadays according to the physical form of bioformulation. Solid carriers commonly are derived from soil materials like peat/plant soil, coal, clays, and lime (Hartley et al., 2004), some are derived from organic materials (saw-dust, composts, charcoal, chitosan and alginate (Bashan et al, 2002; Power et al, 2011), or some are inorganic material like talc, vermiculite, bentonite and kaolin (Smith, 1992). Liquid bioformulation can be produced in broth medium, carbohydrate, mineral or organic oil, emulsions and microbial suspensions. Some examples of carrier-based bioformulation applied on various crops have been listed in **Table 1**.

Currently, different types of carrier material are available but the selection of a suitable one is a must, because it is the carrier’s material that supports the survival of bioagents. The degree of support of carrier material depends upon the nutrient and moisture holding status of the carrier. The high moisture retaining carriers having a low C:N ratio and pH near 7 is considered to be the best for increasing the shelf life of the bioformulation (Arora et al., 2014; Sohaib et al., 2020). Arora et al. (2014) tested the capacity of survival of different carrier materials such as sand, begasse, saw dust, wood ash, and coriander husk and found that higher moisture retaining carrier i.e., Coriender husk which retains 7.5 times moisture is the best for sustaining the bacterial survival. However, another experiment conducted by Sohaib et al. (2020) found that carriers having a low C:N ratio i.e., Compost and Biogas slurry are more effective in increasing the shelf life as well as plant growth and development of wheat over carriers having high C:N ratio. Therefore, the water holding capacity and C:N ratio of the carrier must be taken into consideration for the selection of an effective carrier.

Initially, in 1896 gelatin was first used as a carrier in the commercial production of Nitrogen bioformulation in the United States of America. Later ‘peat’ replaced all carriers and was named as a “gold” carrier until the 1990s (Williams 1984). The success of peat-based formulation can be varied according to the physical state of peat in bioformulation (Solid/powder, pellet, liquid/slurry). In a study, granular peat-based bioformulation greatly enhanced plant growth compared to powder and slurry-based bioformulation (Clayton et al., 2004). Peat in combination with either chitin or chitin-like materials, enhances the biocontrol efficiency of bioformulation along with the growth of microbes and promoted seed germination and plant biomass (Manjula and Podile, 2001). Lignite, charcoal, sawdust, various composts, organic wastes, and vermiculite are the other popular alternatives to peat. Inorganic material like talc-based formulations is very popular in India, as it is economical and easily available. Despite its limitations, this talc-based formulation has shown to be beneficial in various crops as biological control and enhancer of plant growth (Saravanakumar et al., 2009). Moreover, in a comparative study of different materials such as talc, kaolinite powder and bran of wheat, barley, and soybean used as a carrier for *Pseudomonas fluorescens* isolate RRb-11 based fertilizers, talc powder based microbes and has a maximum shelf life of 150 days after storage and is also best to manage bacterial leaf blight disease in rice (Jambhulkar and Sharma 2014). Whereas some *Pseudomonas* strains in peat bioformulation could stabilize for two years at ambient temperature (Georgakopoulos et al., 2002).

Bioformulations with easily degradable high carbon containing carriers like biochar based *Bradyrhizobium japonicum* lead to higher bacterial survival efficiency and better nodulation in soybean (Głodowska et al., 2017). Charcoal-based carrier, i.e., biochar, enhances the survivability of bioformulation and is environmentally benign as they don’t have any hazardous impacts. Another advantage of employing charcoal is that it may be kept without being sterilized owing to its low water content. In addition, Alginate is a nontoxic biodegradable synthetic polymer and is also used in the encapsulation of microorganisms. Alginate-based carrier provides longer shelf life to microbes and provides constant and slow delivery of inoculums to their target site (Bashan, 1986). A study has shown that dried alginate beads could sustain microbial survival for up to 14 years (Bashan and González, 1999). Bioformulations using *Bacillus subtilis* and *Pseudomonas corrugate* in alginate-based formulations produced incredible outcomes compared to charcoal and liquid-based bioformulations (Trivedi et al., 2005).

### 3.2 Adjuvant/adhesives used in bioformulation

Adjuvants/adhesives are natural or synthetic polymers or polysaccharides, polyalcohol derivatives, or caseinate salts that increase the stabilization of microbes, enhance the adhesion potential, help in handling and mixing, and reduce the amount of dust in bioformulation (Jambhulkar et al., 2016; Pedrini et al., 2017). Adhesive application in bioformulation also prevents the dispersion of inoculants during sowing. Nowadays, carboxymethyl cellulose (Zhou et al., 2017), methyl cellulose (Lopisso et al., 2017), gum arabic (Ehteshamul-Haque et al., 2007), pelgel (Ugoji et al., 2006), skim milk (Power et al., 2011), humic acids (Schoebitz et al., 2013), PVP (Polyvinylpyrrolidone) (Surendra and Baby, 2016), glycerol (Anitha et al., 2016) and trehalose (Surendra and Baby, 2016) are generally used as an adjuvant in bioformulation preparation. Carboxymethyl cellulose (CMC) is a non-ionic water-soluble polymer, which is the most common or widely used adjuvant because of its easy availability and cheap economical value. Stimulatory effects of CMC have been demonstrated in various studies for increasing the shelf life and efficacy. Application of CMC supplemented saw dust carrier-based *Rhizobium* inoculants with *M. pruriens* demonstrated fighting against *M. phaseolina* pathogen (Aeron et al., 2012). In one study of chickpea (*Cicer arietinum* L.) seed treatment with CMC based *Pseudomonas jesenii* MP1 and *Rhodococcus qingshengii* S1010 bioformulations results in increased overall crop growth and soil nutrition (Joshi et al., 2019). Gum arabic is a complex polysaccharide, that protects the microbes from desiccation and increases their survivability (Wani et al., 2007). Poly vinyl pyrrolidone is a synthetic polymer that helps the survival of *Bradyrhizobium japonicum* in formulation (Singleton et al., 2002). PVP also protects against desiccation and provides the defense to inoculated microbes against toxic phytochemicals secreted by seed coats during germination. The additional adhesive layering of seeds with superfine calcium salts has decreased seedling mortality and increased plant growth. Here, calcium salts promote plant growth by balancing the acidic nature of the soil (Murata et al., 2008). The use of humic acid as an additive with Ca^2+^ amended alginate-based encapsulation of *Bacillus*, resulted in high bacterial survival and a positive impact on plant growth. The advantage of the inclusion of humic acid in this formulation is its function as a carbon source for the bacteria, which may lead to the survival of microbes during long storage (Rekha et al., 2007).

### 3.3 Adjuvants in liquid bioformulation

Generally, it has been seen that solid carrier-based bioformulations exhibit low shelf life and cannot retain bacterial load during the crop cycle (Chaudhary et al., 2020). To answer this problem, the use of liquid-based bioformulation is a better option. They provide long shelf life to microbes and maintain the survival of bacteria during the whole crop cycle. They also provide temperature and stress tolerance to bioinoculant (Chandra et al., 2018). The use of various adjuvants/adhesives in liquid bioformulations can improve the survival of microbes in a stressful environment, which results in better establishment with host interactions (Mugilan et al., 2011). The amendment of glycerol imparts long shelf life and stress tolerance to *Pseudomonas* against high temperature and desiccation via increased water holding capacity (Taurian et al., 2010). In another liquid bioformulation, *Azospirillum* in 16mM trehalose and phosphate solubilizing strain in 3% PVP maintain very high microbial density (10^8^ CFU/ml) as PVP protect microbes in toxic and stressed circumstances because of their water retention capacity (Surendra and Baby, 2016). Therefore, it can also be concluded that glycerol, PVP or trehalose amended liquid bioformulation can be more reliable and have high potential in the agricultural field. The survivability of microbes depends upon the physio-chemical properties of the carrier. So carriers must be selected based on microbial multiplication and survival during storage and the general method of planting. In summary, each carrier and adjuvant have some advantages and disadvantages. So, the selection of a carrier for bioformulation production is an essential step which majorly depends upon the cost, effectiveness, and need of the grower.

## 4 Types of bio-formulation

Bioformulation is a biologically active component of microbial biomass and its metabolites with the carrier material. It can be used as a plant growth promoting agent, nutrient acquisition, biocontrol, etc., in eco-friendly means (Aamir et al., 2020). The bioformulations can be categorized into solid, liquid, encapsulated, metabolites, and cell-free culture supernatant (Mishra and Arora, 2016; Tewari et al., 2020). Some of the bioformulation categories are mentioned below (**Table 2**).

**Table 2:** Categorization of bioformulations based on their characteristics and carrier.

Main categories	Sub-categories	Characteristics	Carrier used	Reference
Solid	Granular	Dry particles, active ingredients (5 -20%), coarse particles (100 -1000 µm), non-dusty	Wheat granules, corn meal baits, gluten, cottonseed flour, gelatin, sodium alginate, semolina wheat flour, and pesta granules	Tamez-Guerra et al., 1996; Behle et al., 1997; Andersch et al., 1998; Navon, 2000
	Wettable powdered (WP)	50 – 80% powder, 15 – 45% filler, 1 – 10% dispersant, and 3 – 5% surfactant	Wheat bran-sand mixtures, sawdust sand molasses mixture, organic cakes, farmyard manure, talc, charcoal, and flyash	Jambhulkar and Sharma, 2014; Zimdahl, 2018
	Wettable/Water-dispersible granular (WDG)	Small granules, non-dusty, free-flowing, with dry dispersible agents, eco-friendly and readily miscible with water	Water or some other liquids	Mishra and Arora, 2016
Liquid	Suspension concentrate	Non-dusty, measurable and easily pour for spraying purposes	Water, broth, fruit juices, jaggery syrups and polyvinylpyrrolidone (PVP)	Michereff Filho et al., 2009; Singh and Merchant, 2012; Tadros, 2013
	Oil miscible flowable concentrate	Suspension with active ingredients in organic liquids	Organic liquids	Singh and Merchant, 2012; Michereff Filho et al., 2009
Encapsulated	Macro and microencapsulation	Coating of microbial cells within a polymeric material to produce beads, macroencapsulation bead size (mm - cm), microencapsulation bead size (1 – 1000 µm)	Natural polymers i.e. alginate, agarose, chitosan, cellulose, collagen, xanthan, and synthetic polymers i.e. poly(ethylene glycol), polyvinyl alcohol, polyurethane, poly(ether-sulfone), polypropylene, sodium polystyrene sulfate, and polyacrylate poly(acrylonitrile-sodium methallylsulfonate)	González-Ferrero et al., 2020; Wu et al., 2020
Metabolites	–	Bacterial secondary metabolites, act as bioregulator, enhance plant growth, control phytopathogenic attack	Inert carriers i.e. talc, peat, vermiculite, silicates, polyacrylamide beads, charcoal etc.	Confortin et al., 2019; Chatterton and Punja 2009; Onofre-Lemus et al., 2009;
Cell free culture supernatant (CFCS)	–	Cellular supernatant with suitable carrier, higher shelf-life	Talc, charcoal, CaCO_3_	Tewari et al., 2020

### 4.1 Solid bioformulation

After field applications, solid bioformulations provide the protective and nutritive platform for desired microbes. It reduces contamination chances and enhances storage efficiency. It includes granules, powdered, and water-dispersible granular formulations containing active ingredients, binders, and carrier material. Based on applications, the solid bioformulation materials include soil-derived carriers i.e. charcoal, fine clay, turf, organic carriers i.e. sawdust, wheat, soy and oat bran, vermicompost, sewage sludge, animal manure, cork compost and inert carriers i.e. talc, peat, perlite, vermiculite, alginate, bentonite, kaolin, silicates, polyacrylamide beads, charcoal, etc. (Mishra and Arora, 2016). Further, solid bioformulation is characterized by the following:

#### 4.1.1 Granular formulation

The granular bioformulation comprises dry particles with active ingredients (5 – 20%), binder, and granular carrier (Brar et al., 2006). Granules are coarse particles (size 100 – 1000 µm), non-dusty, and without risk of inhalation. Some commonly used granules are wheat granules (Navon, 2000), corn meal baits (Tamez-Guerra et al., 1996), gluten (Behle et al., 1997), cottonseed flour, gelatin, sodium alginate, semolina wheat flour (Andersch et al., 1998), and pesta granules (Wong et al., 2019). Granular bioformulations are quite effective with some limitations, including the inactivation of active constituents in the presence of Ultraviolet light. Wong et al. (2019) reported reduced disease severity (> 43%) of rhizosphere when applied with pesta granules in the roots of Bananas. Researchers have observed that granular bioformulation was superior to peat and liquid carrier in terms of total biomass, nitrogen fixation, and nodule formation under stress conditions (Zaidi et al., 2017). Peat is adaptive, nontoxic, similar to soil, and made of the decomposition of vegetative materials with high water holding capacity (Ceglie et al., 2015). Aini et al. (2019) confirmed that the peat can be used as a carrier for ectomycorrhizal and arbuscular mycorrhizal fungi. While granules have more advantages over peat. Granules contain living microorganisms inside and covering made of calcite, marble, silica, etc, and are easier to handle, transport, and storage. Vermiculite is another type of granule with yellowish-brown material like mica with moisture-retentive properties. It has been used as a carrier for PGP bacteria i.e. *Bacillus* sp. and *Pseudomonas* sp. (Maheshwari et al., 2015).

#### 4.1.2 Wettable powdered formulation

Wettable powdered (WP) formulation consists of 50 – 80% powder, 15 – 45% filler, 1 – 10% dispersant, and 3 – 5% surfactant (Brar et al., 2006). These formulations are readily miscible with water and long shelf life of up to 18 months. Active ingredients impregnate this kind of bioformulation, and after applying water, it can be used as a standard insecticidal spray. WP formulations have some benefits, including uniform distribution of essential gradients, residual control, high holding of active gradients, without sedimentation issues, and fewer skin hazards. WP formulation can be hazardous after inhalation and needs precaution while mixing or agitating vigorously. It is difficult to mix in very hard or alkaline water and clog nozzles and screens. Various herbicides i.e., triazines, phenyl ureas, uracils, and others, have been prepared by WP formulation (Zimdahl, 2018). Wheat bran-sand mixtures, saw dust and molasses mixture, organic cakes, farmyard manure, talc, charcoal, and fly ash are some carriers used in preparations of WP formulation. Talc is an inert material used broadly to study rhizospheric soil bacteria viz. *Bacillus* spp., *B. firmus* (Suryadi et al., 2013), *P. aeruginosa*, *P. fluorescens* (Jambhulkar and Sharma, 2014) etc. While charcoal is free from waxy material, eco-friendly, and can be stored for a long time without sterilization with low water content.

#### 4.1.3 Wettable/water-dispersible granular formulation

Wettable/water dispersible small granules are solid, non-dusty, free-flowing, with dry dispersible agents, which are eco-friendly and readily miscible with water. This formulation has a major role in nematode control and consists of 90% of nematode-based products available in the market (Mishra and Arora, 2016). It bears similar properties to wettable powdered (WP) formulations and can replace those (Ijaz et al., 2019). WDGs have advantages over WP as easy to handle, transport, and mix, seldom clog nozzles, and reduced applicator exposure during mixing and loading. The limitations of WDGs are abrasiveness to sprayers, leaving a visible residue in the container’s bottom, and the requirement of moderate agitation.

### 4.2 Liquid bioformulation

Liquid formulations are aqueous suspensions and consist of biomass suspensions in water, oils or both (Schisler et al., 2004). It contains 10 – 40% microorganisms, 1 – 3% suspender, 1 – 5% dispersant, 3 – 8% surfactant, and 35 – 65% carrier liquid (Brar et al., 2006). Liquid bioformulations are helpful in stabilizing organisms throughout production, distribution, and storage. It protects from abiotic environmental factors and increases persistence. Liquid bioformulations can be categorized as suspension concentrates (Tadros, 2013), oil-miscible flowable concentrate, ultralow volume suspension (Singh and Merchant, 2012), and oil dispersion (Mbarga et al., 2014). The liquid carriers may be water, broth, fruit juices, jaggery syrups, and polyvinylpyrrolidone. The suspension concentrates have been prepared by mixing solid active ingredients with poorly soluble in water and stable to hydrolysis (Tadros, 2013). This mixture is non-dusty, measurable, and easily poured for spray. The oil-miscible flowable concentrate is a suspension with active ingredients in organic liquids. The ultralow volume suspension is used in their respective equipment. This equipment is aerial or ground spray for fine spray purposes (Singh and Merchant, 2012). Oil dispersion formulation comprises one active ingredient suspended in the oil phase and is chiefly used as herbicide and insecticide. NagaChandrabose (2018) has been found efficient liquid bioformulation of *P. fluorescens*, *Purpureocillium lilacinum*, and *Trichodermaviride* against the natural population of root-knot nematode *Meloidogynehapla*. Recently, Prakash and Arora (2020) developed a liquid bioformulation to enhance the growth, nutrient uptake and stevioside content of *Stevia rebaudiana* by using paneer-whey. Moreover, oils of groundnut, pongamia, and sunflower with nutrient broth and water have been used as a carrier, which retains the survival of *B. subtilis*, *Brevibacillus borstelensis*, *Brevibacillus* sp, *Lysinibacillus xylanilyticus*, and consortium (Jayasudha et al., 2018). Hence, liquid bioformulations help to enhance the shelf life of products and act as an excellent carrier to stabilize the bioinoculants throughout production, distribution, and long-duration storage.

### 4.3 Encapsulated bioformulation

The solid and liquid bioformulations have certain limitations like long term storage and viability of microbial spores. In such a scenario, immobilization and encapsulation have improved shelf-life and eased the field application of bioinoculants (Wong et al., 2019; Vassilev et al., 2020). Encapsulation provides controlled release of dynamic target bacterial cells and their metabolites in their rhizospheric environment (Wu et al., 2020) which gives a new strategy for soil microflora improvement and development of sustainable agriculture. Encapsulated bioformulations involve the coating of microbial cells within a polymeric material to produce beads, which are permeable to nutrients, gases, and metabolites for maintaining cell viability within the beads (John et al., 2010). Encapsulated bioformulation protects the active microbial components under unfavorable or environmental stress conditions i.e. mechanical injuries, pH, temperature, biochemical factors, ionic strength, etc. Gelatin, cellulose, starch, and some other polymers have been used in the encapsulation process (Cheze-Lange et al., 2002). There are two types of methods of encapsulation formation i.e. macro-encapsulation and microencapsulation. The macro-encapsulation involves beads of millimeters to centimeters in size, while microencapsulation is 1 – 1000 µm in size. Humic acids have significantly higher viability for encapsulation in certain bacteria. Natural polymers i.e. alginate, agarose, chitosan, cellulose, collagen, xanthan, and synthetic polymers i.e. poly(ethylene glycol), polyvinyl alcohol, polyurethane, poly(ether-sulfone), polypropylene, sodium polystyrene sulfate, and polyacrylate poly(acrylonitrile-sodium methallylsulfonate) have also been distinguished for cell encapsulation (De Vos et al., 2014). Diversity among nitrogen fixing bacteria (NFB) in symbiotic and non-symbiotic associations has revolutionized the crop yield and progress of sustainable agriculture. In this progress, microencapsulation in biofertilizers provides an alternative approach to the development of traditional nitrogen-based fertilizers. NFB and nodule-forming bacteria in association with the nodules of lupine plants of Southern Chile and their microencapsulation by spray drying using sodium alginate: maltodextrin has provided an alternative approach for Nitrogen biofertilizer (Campos et al., 2014). Recently, a novel electrospun microbial composite-based seed coat encapsulation of Canola (*Brassicanapus*) seeds has been developed for its rhizosphere stabilization by using a composite of poly(vinyl alcohol)/poly(vinylpyrrolidone) plasticized with glycerol and the microbial consortium of *Bacillus subtilis* and *Serratia marcescens* (Hussain et al., 2019).

### 4.4 Metabolite bioformulation

Bacteria secrete various secondary metabolites to act as bioregulators, plant growth promoters, and antagonists against phytopathogens (**Table 3**). Moreover, such microbial metabolites are β-1,3-glucanase (Confortin et al., 2019), ACC-deaminase (Onofre-Lemus et al., 2009), Hydrogen cyanide (Olanrewaju et al., 2017), phenazines (Biessy and Filion, 2018), pyrrolnitrin (Pawar et al., 2019), 2,4-diacetylphloroglucinol (Almario et al., 2017), pyoluteorin (Keswani et al., 2020), viscosinamide, tensin, Amphisin (Nielsen et al., 1999), siderophores (Deveau et al., 2016);, pyochelin (Ho et al., 2018), tetracenomycin (Gurusinghe et al., 2019), dialkylresorcinols (Schöner et al., 2015), peptides antibiotics and rhizoxins (Gross and Loper, 2009), mupirocin (El-Sayed et al., 2001), oxyvinylglycines (Okrent et al., 2016), orfamide A and H (Ma et al., 2020), phenazine-1-carboxylic acid (Morrison et al., 2017), furanomycin (Masschelein et al., 2017), brabantamide A (Schmidt et al., 2014), obafluorin (Pu et al., 1994), eruginaldehyde (Ye et al., 2014), safracins (Santos Kron et al., 2020), syringomycins SP22 or SP25 (Bensaci et al., 2011), tabtoxin (Arrebola et al., 2011), syringopeptins (Grgurina et al., 2005), rimid (Matilla et al., 2016), kalimantacin (Thistlethwaite et al., 2017) etc. These metabolites exhibit various properties like antimicrobial activity, insecticidal properties, mobilization of nutrient elements, eliciting plant defense systems, and acting as biosurfactants (**Table 3**). Most of the above said microbial metabolites belong to the secretions of soil rhizosphere microbial communities. Pieces of evidence support that using such a combination of metabolite-producing bacteria as bioinoculants may promote plant growth and enhance agricultural productivity (Morel et al., 2015; De Souza et al., 2017; Santiago et al., 2017; Tewari et al., 2020). The isolation, characterization, and structural elucidation of bioactive microbial metabolites have depended on high-throughput technologies of molecular biology and analytical chemistry i.e. DNA chip, UV-Vis, Ultra-high-performance liquid chromatography – diode array detector – quadrupole time-of-flight mass spectrometer (UHPLC-DAD-QToF-MS), etc. (Gurusinghe et al., 2019; Kwon et al., 2019).

**Table 3:** Overview of sources, target site and properties of microbial metabolites.

SN	Metabolites	Microbial source	Target site	Properties	Reference
1.	β-1,3-glucanase	*B. bassiana*, *P. fluorescens, C. rosea f. catenulata*	Fungi cell wall containing β-glucans	Target insects i.e. *Aproaerema modicella* and fungi i.e. *A. niger*, *Fusarium* sp. *Pythium* sp.	Confortin et al., 2019; Chatterton and Punja 2009
2.	ACC-deaminase	*Burkholderia* spp.	Host plant secreted ethylene	Efflux of plant ACC, plant growth promoting under stress conditions like flooding, saline condition, drought etc.	Onofre-Lemus et al., 2009
3.	Chitinase	*C. rosea* f. *catenulata* *T. harzianum*, *A. album*,	Fungal cell wall	Target fungi i.e. *Aspergillus niger, Fusarium* sp. *Pythium* sp.	Chatterton and Punja 2009
4.	Hydrogen cyanide (HCN)	Bacterial genera i.e. *Rhizobium*, *Pseudomonas*, *Alcaligenes*, *Bacillus*,	Inhibit cytochrome c oxidase	Antimicrobial against fungi and bacteria, mobilization of elements from rock forming i.e. phosphate	Olanrewaju et al., 2017
5.	Phenazines	Pseudomonads i.e. *P. chlororaphis*, *P. fluorescens*, *B. linens*, *B. cepacia*, *M. amazei*, *P. agglomerans*	Interact with cell membrane, uncoupling of oxidative phosphorylation, the generation of ROS	Broad spectrum antibiotic properties, inhibit the growth of eukaryotic plant pathogens including fungi and nematodes	Yu et al., 2018; Biessy and Filion, 2018
6.	Pyrrolnitrin	*Pseudomonas pyrrocinia*, *Pseudomonas* spp., *Burkholderia* species	Target terminal electron transport system	Natural antifungal antibiotics	Tripathi and Gottlieb 1969; Pawar et al., 2019
7.	2,4-diacetylphloroglucinol	Fluorescent Pseudomonads	Protein gradient across the mitochondrial membrane	Elicit plant defences through induced systemic resistance, modulation of plant hormonal balance by acting as auxin-mimetic compound	Almario et al., 2017
8.	Pyoluteorin	Pseudomonads	Unknown	Control soil-borne diseases	Keswani et al., 2020
9.	Viscosinamide	*Pseudomonas fluorescens* DR54	Tightly coupled to cell proliferation	Biosurfactant, Antifungal properties against *Pythium ultimum* and *Rhizoctonia solani*	Nielsen et al., 1999
10.	Tensin, Amphisin	*Pseudomonas fluorescens* 96.578	Unknown	Provide site for bacterial attachment, Antifungal properties against *P. ultimum* and *R. solani*	Nielsen et al., 1999;
11.	Siderophores	Pseudomonads	High-affinity iron-chelating compounds	Provide microbial ability to obtain iron from the environment and exhibit antagonistic activity	Deveau et al., 2016; Rezanka et al., 2019
12.	Pyochelin	*Pseudomonas aeruginosa*	ISR with ROS, work with Fe–pyochelin and pyocyanin synergistically	Antimicrobial properties against *Pythium* sp., *Xanthomonas* spp., and other phytopathogens	Ho et al., 2018
13.	Tetracenomycin	*Streptomyces glaucescens*, *Acinetobacter* sp.	Cell membrane	Aromatic polyketide antibiotics	Gurusinghe et al., 2019
14.	Dialkylresorcinols	*Pseudomonas* spp.	Cell-cell communication molecule	Antimicrobial properties against Gram-positive bacteria, mycobacteria, yeasts, and fungi	Schöner et al., 2015
15.	Peptides antibiotics	Fluorescent *Pseudomonas* spp.	Cell membrane	Antifungal agent, biosurfactant	Gross and Loper, 2009
16.	Rhizoxins	*Rhizopus microsporus*, *Pseudomonasfluorescens*	Binding to β-tubulin, thereby interfering with microtubule dynamics	16-membered polyketide macrolides exhibit significant phytotoxic, antifungal, and antitumoral activity	Gross and Loper, 2009;
17.	Mupirocin	*P. fluorescens*	Blocks protein synthesis in bacteria	Polyketide antibiotic exhibits antibacterial activity against Gram positive bacteria	Capobianco et al., 1989; El-Sayed et al., 2001
18.	Oxyvinylglycines	*Pseudomonas* spp.	Inhibit cellular enzymes that require pyridoxal phosphate (PLP) as a co-factor	Naturally produced non-proteinogenic amino acids	Okrent et al., 2016
19.	Orfamide A	*Pseudomonas fluorescens* Pf-5, *P. protegens*F6	Target β-glucan synthesis	Cyclic lipopeptide, exhibit antifungal and insecticidal properties	Jang et al., 2013; Oni et al., 2019
20.	Orfamide H	*Pseudomonas protegens* CHA0	Target β-glucan synthesis	Inhibiting the aspersoria formation of the fungus *Magnaporthe oryzae*	Oni et al., 2019; Ma et al., 2020
21.	Phenazine-1-carboxylic acid	*Pseudomonasfluorescens* (LBUM636), *P. aeruginosa* LV	Act on exopolysaccharide formation, distort and damaged fungal hyphae	Antifungal activity against *Phytophthora infestans*, *Botrytis cinerea*	Morrison et al., 2017; Simionato et al., 2017
22.	Furanomycin	*P.* fluorescens SBW25, *S. threomyceticus*	Isoleucyl-tRNA synthetase	Antibacterial properties	Trippe et al., 2013; Masschelein et al., 2017
23.	Brabantamide A	*Pseudomonas* sp.	Cell wall	Antibacterial, antifungal and anti-oomycete activity	Schmidt et al., 2014
24.	Obafluorin	*P. fluorescens* (SC12936)	Unknown	Antibacterial properties	Pu et al., 1994
25.	Aeruginaldehyde	*P. fluorescens*, *Burkholderia cepacia*	Unknown	Antifungal properties	Ye et al., 2014
26.	Safracins	*P. fluorescens*	DNA	Antagonist activity against *Erwiniaamylovora* in apple flower	Santos Kron et al., 2020
27.	Syringomycins SP22 or SP25	*P. syringae*	Lipid of cell membrane	Antifungal against *Saccharomyces cerevisiae* and *Candida albicans*	Bender et al., 1999; Bensaci et al., 2011
28.	Tabtoxin	*P. syringae*	Glutamine synthetase	Antibacterial and phytotoxic properties	Arrebola et al., 2011
29.	Syringopeptins	*P. syringae*	Cell membrane	Antibacterial, antifungal, Biosurfactant properties	Scholz-Schroeder et al., 2001; Grgurina et al., 2005
30.	Andrimid	*P.fluorescens*, *Pantoea agglomerans*	Acetyl-CoA carboxylase	Antibacterial properties	Matilla et al., 2016; Liu et al., 2008
31.	Kalimantacin	*Pseudomonas* sp.	FabI	Antibacterial properties	Thistlethwaite et al., 2017

### 4.5 Cell free culture supernatant bioformulation

The secreted products in the form of enzymes, toxins, and other metabolites from desired microbial cells can be used to prepare CFCS bioformulation. The CFCS bioformulation is prepared by separating the supernatant from the cell pellet by centrifugation and passing through a 0.22 µm filter and mixing with a suitable carrier (Tewari et al., 2020). Multiple studies have significantly isolated and implemented the CFCS to enrich soil fertility and crop improvement. Patel and Thakker (2020) have assessed the amount of soluble phosphate from the CFCS while evaluating the mineral weathering efficiency of *Streptomyces nanhaiensis* YM4, the rhizospheric fungi of the millet crop. Moreover, in studying biocementation process of soil by calcite and aragonite, *Citrobacter freundii* and *Pseudomonas azotoformans* have been reported highest extracellular urease activities i.e. 45.5 ± 3.4 and 54.9 ± 3.5 U/ml, respectively. The study confirms that cell-free supernatants of *C. freundii* and *P. azotoformans* have participated in the precipitation of CaCO_3_ from the cementation solution of urea and CaCl_2_ (Abdel-Aleem et al., 2019). Manhas and Kaur (2016) have reported the biocontrol potential of *Streptomyces hydrogenans* and, cell and culture supernatant against *Alternaria brassicicola*, the causal agent of black leaf spot and damping-off of seedlings of crucifers. Recently, Kaur et al. (2019) have worked on biocontrol and plant growth promoting properties of *Streptomyces* sp. MR14, the soil actinobacteria, concluded its role of supernatant and extract in suppressing *Fusarium* wilt disease caused by *Fusarium moniliforme* in tomato plants. In a similar study, *Bacillus amyloliquefaciens* LZN01 showed antagonistic properties against *Fusarium oxysporum* f. sp. *niveum*, which was examined by functional components of CFCS from *B*. *amyloliquefaciens*. CFCS had shown damage to cell membrane integrity, which was further confirmed by confocal laser scanning microscopy. The major metabolites in CFCS were identified as myriocin, sphingofungin E, sphingofungin F, 3-methyl-2-oxovaleric acid, gabapentin, and sphingofungin C (Xu et al., 2019). Further, Silambarasan et al. (2012) have explained the antagonistic properties of CFCS of Actinobacteria isolates from Ratnagiri hills, Tamil Nadu, against *Bacillus subtilis*, *Klebsiella*, *B. cerus*, *Staphylococcus aureus*, *Escherichia coli*, *Curvularia* sp., *Candida albicans*, *C. trophicalis*. CFCS bioformulation has been observed with higher shelf life than living cells for plant growth promotion (Tewari et al., 2020). Hence, CFCS can provide the scenario for next-generation bioformulations by enhancing crop productivity and the development of sustainable agriculture.

## 5 Factors affecting the efficacy of microbial bioformulation

The efficiency of microbial formulations can be altered by various biotic and abiotic factors. These factors affect the acclimatization, viability, activities, and overall performance of microbial formulation. Some key factors that can impact microbial formulation efficiency are listed below (Mawar et al., 2021; Rojas-Sánchez et al., 2022):

➢ **Strain Selection:** The selection of appropriate microbial strains is vital, as different strains have varying abilities to thrive in different environmental conditions and they only perform desired functions at their best in their loving environment conditions.
➢ **Carrier:** The choice of carrier materials or additives in the formulation directly influences the protection, delivery, and release of the microbes. These materials should be selected to enhance microbial survival and activity.
➢ **Storage Conditions:** Proper storage conditions, including temperature, humidity, and packaging, are critical to maintaining the viability of the microbes in the formulation.
➢ **Shelf Life:** The shelf life of the formulation can significantly impact its efficiency. Microbial formulation having shorter shelf lives may require more frequent application, while longer shelf lives can reduce the need for frequent reapplication.
➢ **Environmental competition:** The ability of microbes to adhere to surfaces and colonize their intended habitat is crucial because microbes in formulations may face stressors such as UV radiation, chemical exposure, and competition with native microorganisms. Interactions with native microorganisms or other introduced strains can affect the performance of the formulated microbes.
➢ **Application Method:** The method of application, whether through spraying, irrigation, injection, or other means, can impact the distribution and effectiveness of the formulation in the target area.
➢ **Environmental Conditions:** External environmental conditions, such as seasonal variations and climate changes which determine the biotic and abiotic factors (pH, Temperature, salinity, soil type, microbiota, etc.) of such regions can affect the efficiency of microbial formulations.
➢ **Quality Control:** Rigorous quality control measures during the manufacturing process are critical to ensure consistency and reliability in microbial formulations because contamination of any foreign microorganisms greatly affects bioformulation efficiency.
➢ **Genetic Stability:** In some cases, the genetic stability of the microbial strains in the formulation should be considered to ensure that they maintain their desired traits over time.

Apart from above mentioned factors, numerous other factors are also responsible for influencing the efficiency of microbial formulations. Optimizing these factors based on the specific application and environmental conditions is essential for maximizing the working efficiency of microbial formulations.

## 6 Role of bioformulation

Plant growth promoting microorganisms (PGPM) are those beneficial microbes that help in plant’s growth and development through protection from biotic and abiotic stresses and by maintaining nutrient availability (Upadhayay et al., 2022a; Upadhayay et al., 2022b; Khan et al., 2020b; Khan et al., 2022). Therefore, the implementation of PGPM as a microbial-based formulation is the current time to ensure high crop productivity with better nutritional values of plants and maintain the high nutritional status of soil (Geetha and Balamurugan, 2011; Accinelli et al., 2018).

### 6.1 Enhancer of crop yield and nutritional quality

**Figure 2:**
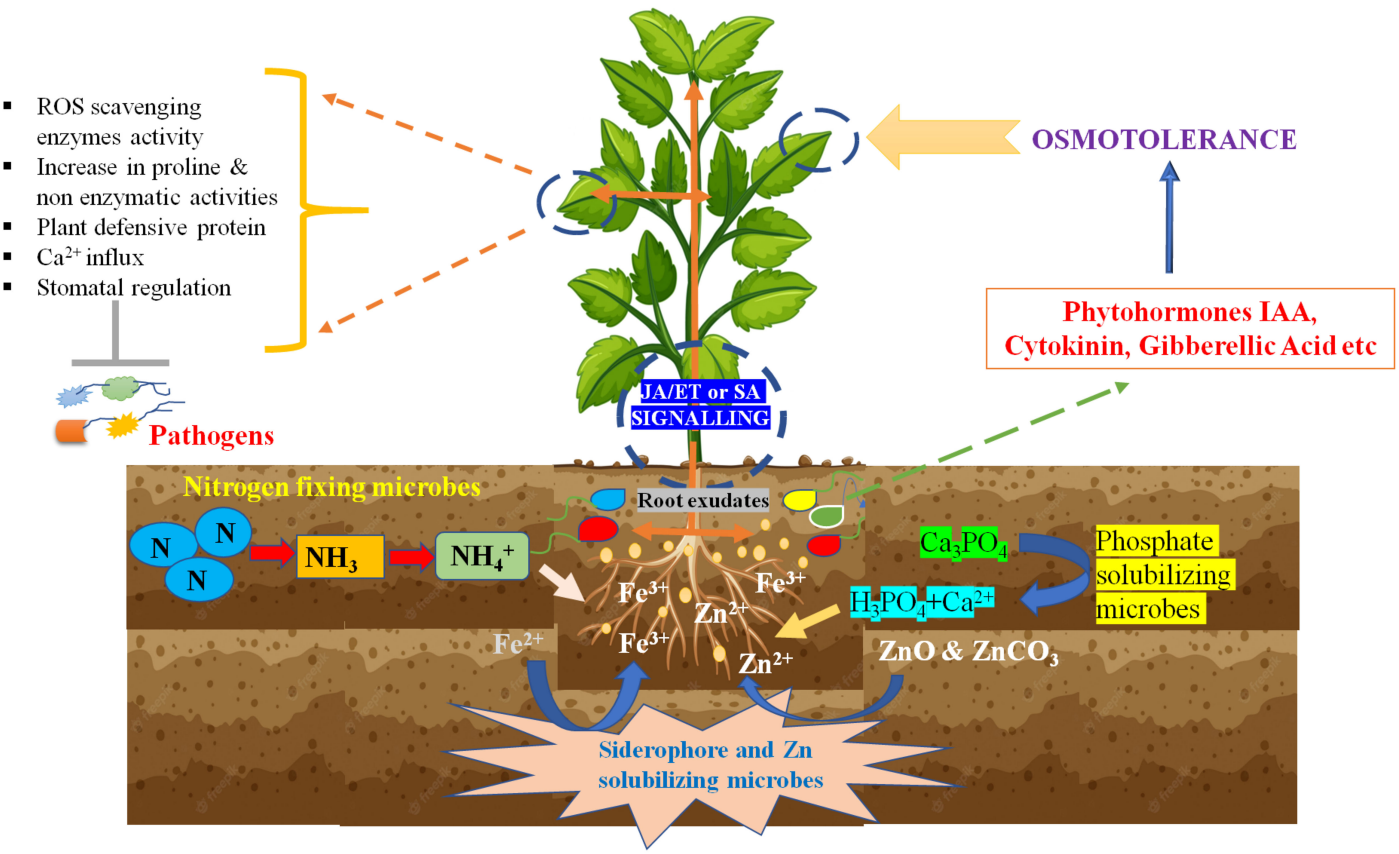
Plant beneficial strategy adopted by plant growth promoting bacteria.

The main application of biofertilizers in agriculture is to ensure food security and the nutritive value of plants for the good health of consumers like humans. After the green revolution, the continuous use of chemical fertilizers was able to fulfill food quality, but it is diminishing the nutritional value of plants and soil. Nitrogen (N), phosphorus (P) and Potassium (K) are essential macronutrients for proper plant growth and act as major limiting factors in terms of crop production as these elements play a vital role in plant metabolism, growth, and development. N, P, and K are present in different forms in soil, but the plants do not take the majority forms (Khan et al., 2019). Hence, most of the soil land in the entire world lacks plant-available nutrients (Karamesouti and Gasparatos, 2017). Therefore, in agriculture practice, the use of chemical fertilizers to increase the NPK content in soil increased, resulting in the leaching of excessive minerals into the soil environment. Plants uptakes nitrogen, phosphorus, and potassium through their roots from the soil, so the application of N-fixation bacteria, phosphate solubilizing bacteria (PSB), and potassium solubilizing bacteria as biofertilizers will increase the available NPK in soil and influence the plant nutritional status along with yield (**Figure 2**). “BioGro” inoculant is a mixture of microbial strains isolated from rice crop soils. The application of this inoculant increases the grain yield and nutrients like N and P content in rice (Nguyen et al., 2017). Colla et al. (2015) reported a significant increment in the growth of shoot, root biomass, and leaves number by 23%, 64%, and 29%, respectively, and an increase in yield (8.3% to 32.1%), depending on the growing season and high nutritional grain quality along with enhancement in protein, K, P, Fe, and Zn concentrations after direct treatment with consortium of arbuscular mycorrhizal (AM) fungi (*R. intraradices* and *F. mosseae*) and *T. atroviride* as compared with untreated. The seed inoculation with the liquid formulation of *Pseudomonas flouorescens* increased the plant growth, biomass, and grain yield, and reduced the recommended dose of N fertilizer in maize (Sandini et al., 2019). A study to identify the best combination of bioformulation and chemical fertilizers for maximum chickpea production in hilly areas found that bioinoculants (N-fixers and PSB) with 20 Kg N/ha urea concentration resulted in high crop yield in chickpea and enhanced the rhizosphere and soil nutrition in comparison to alone biofertilizer, chemical fertilizer, and untreated control, as bioformulation increased the survivability of microbes (Joshi et al., 2019). This combinational approach for applying bio and chemical fertilizer to improve production with economic efficiency was also found applicable in sugarcane (Pereira et al., 2018). These studies showed that the correct combination of appropriate doses of chemical and biofertilizers could boost plant growth, which will help reduce the amount of chemical fertilizers.

### 6.2 Role as biocontrol agents

Bio-control agents (BCA) and inducers of induced systemic resistance (ISR) have been widely studied to reduce the use of chemical fungicides in agriculture crops. In most cases, BCA can control plant pathogens directly or indirectly by developing a non-physical relationship with host-pathogen (**Figure 2**). Another way to prevent the plant from biotic stresses is the competition for micronutrients and space to colonize and survive in the rhizosphere (Upadhayay et al., 2021). BCA colonization at pre-empty infection sites allows them to consume available plant resources and leaves the pathogen for nutrient and space scarcity. In a study of Lindow (1987), plant foliar colonization of *Pseudomonas syringae* strain on pear plants resulted in less infection caused by *Erwinia amylovora* than untreated plants.

Another way to control plant infection against pathogenic microorganisms and insects is to induce an Induced systemic response (ISR) defense system in plants (Pieterse et al., 2014). *Bacillus* spp are reported to produce cyclic lipopeptide compounds that result in plant ISR mechanism elevation through jasmonic acid (JA)/ethylene and salicylic acid (SA) pathways against phytopathogens. Chitin amended talc-based bioformulation of *Pseudomonas fluorescens* Pf1 reduced the disease effect of Macrophomina root rot in Moongbean by inducing the expression of the defense-related proteins and phytochemicals accumulation at the site of infection, which decreased the colonization of pathogens in the root (Saravanakumar et al., 2007). In this study, chitin amendment increased the growth and survival of chitinolytic microbes through acct as a carbon source in bioformulation (Bell et al., 1998).

Singh et al. (2014) found that seed coating of chickpea with a bioformulation using gum arabic as an adjuvant led to higher plant growth and an elevated amount of phenolic compounds in fungal pathogen *Sclerotium rolfsii* infected chickpea, in comparison to untreated control and single inoculations. Similarly, Saravanakumar et al. (2007) studied a mixture of three *Pseudomonas fluorescens* Pf1, TDK1, and PY15 strains to reduce the rot disease in rice with an increase in grain yield (Saravanakumar et al., 2007). In both studies, these consortia led to the activation of the plant host defense mechanism by elevating the level of defense-related enzymes, proteins, and phenolic content in the plant, which causes the ISR mechanism activation in the host to deal with biotic stresses. While in another application of *Trichoderma* strains with two synthetic fungicide agents (acibenzolar-S-methyl and thiamethoxam) decreased disease indices of phytopathogen *Pyrenophora tritici-repentis* in wheat by inducing plant defense system and activating pathogenesis-related enzymes which directed for ethylene signaling (Perelló and Bello 2011). The combination of microbial-based bioformulation with chemical compounds has resulted in more growth and caused less disease occurrence, so the use of the biological and chemical combinatorial approach for healthy plant and crop production will reduce the fungicide application. There is a robust future for new development and research in applying multi-strain carrier-based bioformulation in agriculture to manage biotic stresses.

### 6.3 Controlling abiotic stress

The use of microbial bioformulations is often seen as a viable alternative to improve the crop yield under different abiotic pressures (Singh et al., 2021). Abiotic stress like drought, waterlogging, low or high temperature, salinity stress, and deficient or excessive mineral content negatively influence plant growth, yield, and nutritional quality of seeds. Recently, a research study documented improved cowpea’s biomass and crop yield under water-deficient conditions following treatment with silicon dioxide and starch-based- *P. putida* bioformulation (Rocha et al., 2019b). The study of Sohaib et al. (2020) reported that a bacterial consortium promotes high nitrogen and phosphorus content in straw and grains with better wheat plant growth and crop productivity by mitigating the salt stress and reducing ethylene production in organic compost biogas slurry based carrier bioformulation. Accelerated ethylene production is known to occur in stress conditions and induce senescence by degrading chlorophyll pigments, mineral misbalancing, and inhibiting protein synthesis under salinity stress. This result was also supported by previous research that highlighted the application of ACC deaminase containing bio-inoculants prevented ethylene’s output, which protects the plant from senescence (Zahir et al., 2011). The above-mentioned carrier-based bioformulation surges the survival of the above bacterial consortia until three months, which is best to protect the wheat plant. The same kind of effect was also reported by using PGPB like *Pseudomonas fluorescens* YsS6, *Pseudomonas migulae* 8R6 in peat-based bioformulation in tomato plants (Ali et al., 2014), and application of liquid-based alone or combination of different ACC deaminase producing microbes UW3 (*Pseudomonas* sp.) and UW4 (*P*. sp.) rhizobacterial isolates CMH3 (*P. corrugata*) in both barley and oats under high salt stress (Chang et al., 2014). Under abiotic stress, plant’s survival mechanisms induce through complex signaling pathways, which remarkably enhance by PGPR through the array of mechanisms (Wang et al., 2019). Under stress, plant activates signaling pathways with sensors, receptors, and ion channels. Specific protein kinases, like AtHKT1 in *Arabidopsis thaliana*, detect signals, triggering downstream gene activation via secondary messengers like reactive oxygen species and inositol (Gupta et al., 2022). These messengers induce calcium oscillations, driving stress-responsive protein formation (Ali et al., 2017). In a study, *Bacillus subtilis* priming was reported to modulate the HKT/K+ transporter gene (HKT), improving the K+/Na+ ratio by reducing Na+ uptake (Zhang et al., 2008). In another study, *Pseudomonas fluorescence* and *P. putida* regulate the At3g57530 gene, impacting calcium and calcium-dependent protein kinases (CDPKs). Rhizobacteria offer drought resilience through RIDER (Rhizobacterial-Induced Drought Endurance and Resilience). RIDER involves PGPR-induced changes like producing phytohormones, exopolysaccharides, cyclic metabolic pathways, and reinforcing antioxidant defenses with compounds like amino acids, polyamines, sugars, and heat shock proteins (Saharan et al., 2022). Additionally, the *Piriformospora indica* fungal endophyte was also found to enhance drought resistance by upregulating antioxidant enzymes, drought-related genes, and CAS mRNA levels in stressed leaves (Sun et al., 2010).

In a research endeavor, chickpea seeds were subjected to an experimental treatment involving the use of sodium alginate and CaCl_2_ as carriers for *Paenibacillus lentimorbus* B-30488. This treatment led to a notable proliferation of beneficial bacteria in the soil and the formation of biofilms. Subsequently, this enhanced bacterial activity played a pivotal role in improving the chickpea plants’ resilience to drought stress by positively modulating their physiological responses to dehydration (Khan et al., 2011). Use of sodium alginate and calcium chloride increases the biofilm production and better seed attachment in this bioformulation and leads to overcoming the drought effect in plants. So further, these bioformulations may also be used in the phytoremediation of marine soils.

## 7 Delivery methods of bio-formulation

**Figure 3:**
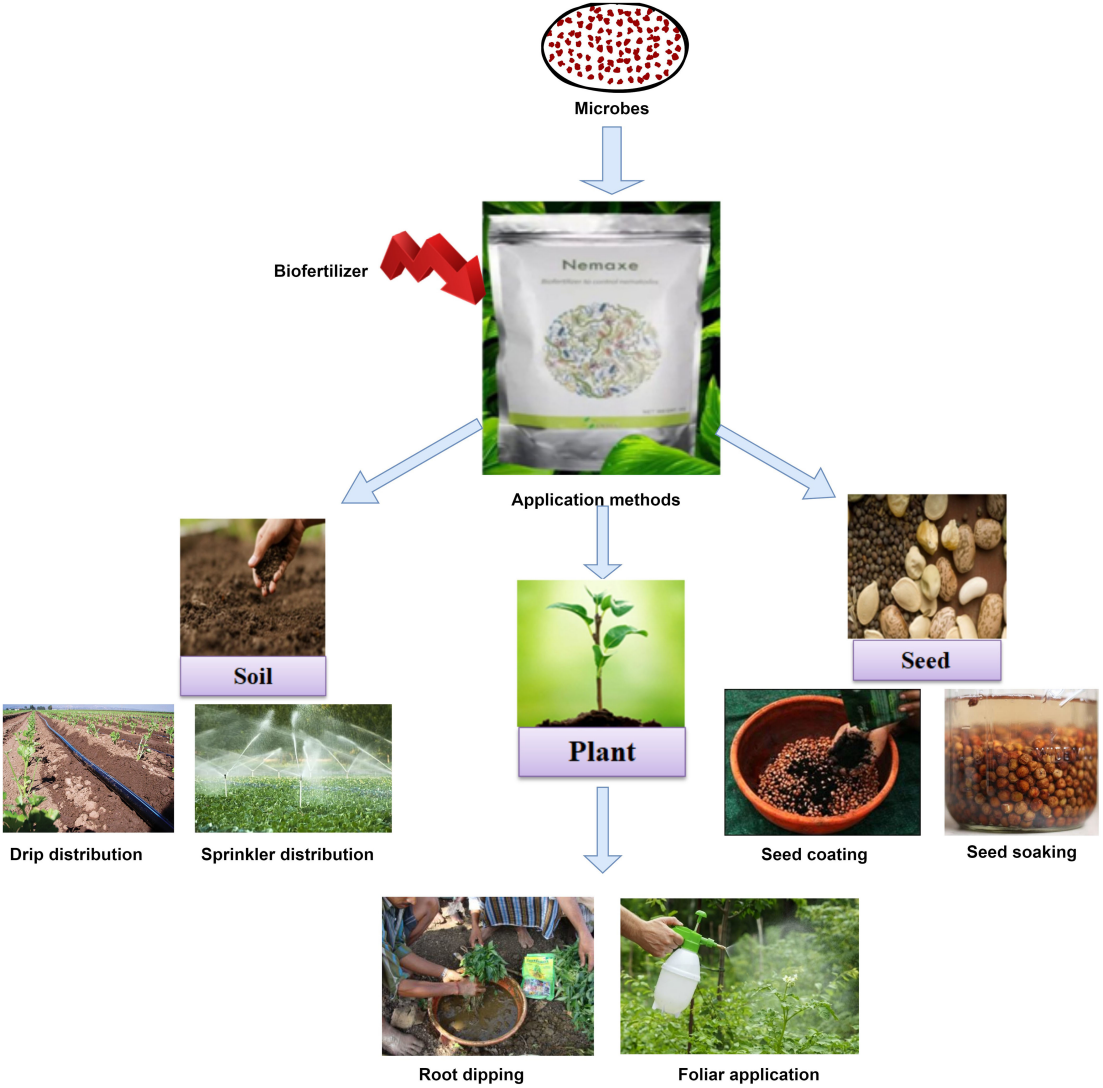
Bioformulation delivery methods for different plant parts.

There are just a few methods for applying microorganisms to crops because of limitations during bioformulation preparations. Nowadays, various devices for micro-based fertilizers application are available, such as rotating drums, mixers, and sprayers, which vary from industrialized to field, affecting bioinoculant application cost and labor time. Bio-formulations are commonly applied in three ways, 1) soil inoculation (direct soil treatment), 2) plant treatment (seedling/root dipping/foliar spray), and 3) through seed coating or treatment of seed (seed soaking) (Mahmood et al., 2016; **Figure 3**). Each method has some advantages and drawbacks, depending upon the amount of inoculant used, equipment requirement, cost, and treatment area (Bashan et al., 2014). The application methods mainly depend on the type of cultivated crop, working efficiency of bioformulation, and types/medium of formulations used (Bejarano and Puopolo, 2020).

### 7.1 Soil treatment

This method is more convenient for farmers because it takes less time for large areas and protects small or fragile seeds from damage, but it is expensive and generally needs a very high amount of inoculants. Soil treatment is used when many bacterial species are being applied to the soil, which enhances the probability of inoculant interaction with the rhizosphere, thus improving overall plant growth. Soil can be treated by solid, liquid, or encapsulation-based bioformulation (Malusa et al., 2012; Bashan et al., 2014). Granular forms of carriers like peat, charcoal, perlite, or other soil material have shown good effect in soil inoculation. Powder, slurries, and liquid inoculants have also been effectively added directly to the soil. In this approach, bioformulation is spread on the top surface of moist soil of the field before sowing using granular applicators, hand or mechanical sprayers. Soil treatment can also be done in the standing crop, but bioinoculants could not be distributed uniformly during these circumstances. Soil treatment is more beneficial when dealing with soil-borne phytopathogens as they protect plants from preoccupying sites. This approach has limitations in large application areas due to cost, large quantities of inoculants, and the requirement of specific equipment (Vosátka et al., 2012).

### 7.2 Plant treatment

It is the direct application of biofertilizer to plants via two methods, either through root dipping or foliar spray. In this way of treatment, we can inoculate more inoculum or a concentrated amount of microbes as it allows multiple applications of bioformulations. In foliar spray, wettable or liquid bio-formulations are usually added to foliar sections of plants with the use of spray equipment which varies from hand to aircraft like mechanical equipment to combat above-ground plant pathogens, especially foliar pathogens, and provide nutrition to plants. A suppression in disease caused by *Sclerotinia sclerotiorium* in canola plants was reported after the foliar application of liquid bioformulation containing consortium of *Pseudomonas chlororaphis* (PA-23), *Bacillus amyloliquifaciens* (BS6 and E16) and *Pseudomonas sp* (DF41) (Fernando et al., 2007). For foliar spray, a major disadvantage is the need of large amount of microbial inoculant, which may be expensive, laborious, and treatment timing as it is limited to low environment temperature, high relative humidity, and turgid leaves during application (Bejarano and Puopolo, 2020). Root dipping of rice seedlings in talc-based bioformulation of *Pseudomonas fluorescens* suspension prior to transplantation reported a decrease in bacterial leaf blight of rice (Jambhulkar and Sharma 2014). This method decreases disease incidences because of previous inoculants colonization in the rice rhizosphere, which primarily avoids the development of the host-pathogen partnership. The root dipping method’s main disadvantage is the preparation of the nursery, which is a mandatory, laborious, and time-consuming procedure (Adholeya et al., 2005; Mahmood et al., 2016).

### 7.3 Seed inoculation

It is the most common bioinoculant application method as it needs a relatively low quantity of bio-formulation, which is widely used in a variety of cereals and legumes (Woomer et al., 2014). Seed inoculation or seed treatment delivers PGPM directly to the rhizosphere of the target plant, or in the case of an endophyte, these microbes enter the plant itself, which helps to develop an intimate plant-microbe interaction (Philippot et al., 2013). Seed treatment may be done using a number of methods, such as seed soaking, seed coating (seed dressing, pelleting/encrusting, film coating, slurry coating), and bio-priming, based on the size, shape, weight of treated seed and equipment availability (Joshi et al., 2019; Rocha et al., 2019a). In brief, seed coating is generally done by making a slurry of carrier-based bioformulation, with or without adjuvant, followed by uniform mixing of slurry onto the seeds, and further drying creates a thin layer of bio-inoculants over seeds (Choi et al., 2016). These inoculants can be applied with seeds by hand, low cost spinning drums, wide dough or cement mixers or hydraulic machines or automated seed coaters (Schulz and Thelen, 2008). Drying could also be performed either by natural air drying or using drying equipment. During liquid bioformulation application, inoculants are sprayed directly on seeds, followed by drying. Another advantage of seed inoculation is that it can also be used to modify seed characteristics (shape, size, weight, etc.) to make it easier to manage seed sowing and supply effective bio-inoculants to seeds (Halmer, 2008). Apart from the many advantages (such as low amount of inoculants, cost effective, fast, ready to use product), seed inoculation through bioformulation also has several drawbacks. The main drawbacks of seed inoculation are poor survival or reduced shelf life of active microbes in bioformulation and less inoculant coating over the small seed (due to lesser surface area). Sometimes seeds may be destroyed during the inoculation process, which prevents the germination of the seed. In some cases, the seed coat can be lifted out of the soil during germination, causing the death of the bacteria. So, the choice of inoculation method depends on the equipment available, the size and shape of the seed, the delicacy of seed coat and cotyledon, and the comfort and cost-effectiveness for the farmer (Deaker et al., 2004).

## 8 Biosafety issue and risk assessment of microbial bioformulations

Biosafety encompasses a set of procedures aimed to prevent biological risks to both humans and the environment. There is substantial literature providing detailed guidelines for handling microorganisms of various biosafety levels. Biosafety levels (BSL) are used to indicate the minimum safety practices recommended for handling different risk-groups of microorganisms. At BSL-1, microorganisms pose low individual and community risks as they are non-pathogenic. BSL-2 microorganisms present moderate individual risk and low community risk, while BSL-3 microorganisms carry high individual risk but still have low community risk. On the other hand, BSL-4 microorganisms are highly pathogenic and represent significant risks to both individuals and communities (Emmert and ASM Task Committee on Laboratory Biosafety, 2013). In the context of organic farming, beneficial microorganisms are being used as biofertilizers in agriculture. Due to existing policy restrictions and selection of the nonpathogenic microorganism belonging to the BSL1, the development of biofertilizers primarily relies on wild-type microbes (bacteria and fungi), which are predominantly sourced from soil and plants rather than human and animal hosts (Bach et al., 2016). But in the current fast research, scientists are focusing only on the beneficial traits of isolates while the pathogenicity is being neglected through paying little attention to characterizing these strains at the species level or studying their pathogenicity before large-scale field applications and by imagining that the bacteria is being isolated from natural source like soil and water and therefore it would be nonpathogenic. In a consequence of this, some of using biofertilizers are found to have belonged to BSL-2 microorganisms and have been shown to behave as opportunistic pathogens, posing risks to both the environment and human health. Apart from this, efforts to improve isolation and selection techniques have led to the discovery of novel genera and species with potential as biofertilizers. However, the lack of reference strains for pathogenicity comparison and the potential presence of closely related strains in hospital environments raise concerns about the safety and applicability of these novel isolates for commercial distribution (Uzcátegui-Negrón et al., 2011). The application of these biofertilizers often results in their proliferation, making them the dominant bacteria. As a consequence, they interact with non-target plants, causing alterations in the composition and prevalence of species, and in some cases, leading to a reduction in local plant biodiversity (Kardol et al., 2007). Moreover, extensive microbial activities of such opportunistic pathogens, including the production of antibiotics or growth-regulatory substances, can significantly impact the local microbial community, affecting the composition and prevalence of beneficial and harmful bacteria in the ecosystem. This, in turn, may lead to disruptions in nutrient cycles and changes in plant biodiversity. Therefore, the use of actinobacteria in agriculture or biotechnology requires rigorous precautions since some of these isolates are associated with life-threatening diseases (Gneiding et al., 2008). Similarly, even well-known PGPRs like *Arthrobacter agilis* can be a cause for concern due to the potential impact of volatile blend emissions or the production of certain substances. Furthermore, *Arthrobacter oxydans* (strain CF39) has been consistently identified in clinical samples, indicating its potential as an opportunistic pathogen (Mages et al., 2008). In the case of arbuscular mycorrhizal (AM) fungi, once considered mutualistic fungal symbionts beneficial to plants, but it was found that they could also be deleterious to their host plants due to competition, leading to changes in plant growth and overall ecosystem dynamics (Bever, 2002).

To prevent the potential pathogens in the environment, even when the PGPR appears as safe, it is highly recommended to conduct in-depth characterization and validations of PGPR strains under controlled conditions before field application. Rather than solely depending on the 16S rRNA gene sequence or any other traditional identification methods, it is crucial to adopt contemporary interdisciplinary tools and a polyphasic approach to comprehensively assess the identification, risk assessment, and ecological significance of each strain. Whole genome sequencing is suggested as a cost-effective and efficient approach to obtaining comprehensive phylogenomic information about isolates, including taxonomic relationships. Moreover, molecular identification and characterization of virulence-related genes can assist in assessing the safety of novel bacterial isolates. Standardization of methodologies and information sharing will aid in the selection of suitable microbes as next-generation bacterial inoculants. The use of the Environmental and Human Safety Index (EHSI) can help catalog isolated strains for PGP and compare them with recognized PGPRs with known pathogenic or deleterious effects on the ecosystem. By adopting the Precautionary Principle and incorporating the Environmental and Human Safety Index, we can make informed decisions to minimize potential risks associated with the use of bacterial inoculants and ensure environmental and human safety (Vílchez et al., 2016).

## 9 Challenges and limitations in utilizing microbial formulation

In recent years, there has been a growing interest in harnessing the power of beneficial soil microorganisms for the production of biofertilizers, aimed at boosting plant productivity. This approach has witnessed significant successes, yet it is not without its set of challenges and constraints. The complexities of replicating their positive effects on plants under ever-changing environmental conditions at field conditions pose a primary hurdle. Furthermore, there is a need to raise awareness within farming communities about the scientific methods of applying microbial bioformulations in the field and the ecological importance of these microbial formulations. Education and outreach efforts are crucial to foster their adoption and successful application. Ethical concerns may also arise, particularly when considering the use of genetically modified microorganisms or non-native species in these formulations. The acceptance of such practices within society can play a pivotal role in their adoption. Additionally, the existing native soil microorganism populations can present significant barriers to the successful implementation of these inoculants. The consistency of microbial biofertilizers across diverse environmental conditions and crop types is not guaranteed. Selecting the right microbial strains for specific agricultural contexts can be a challenging task. Moreover, the efficacy of these strains can vary based on factors like soil type, temperature, pH, and moisture levels. Another limitation is the limited shelf life of microbial formulations. Over time, the viability of microorganisms in these formulations can diminish, reducing their effectiveness in the field. To maintain the consistency and effectiveness of these products, rigorous quality control during production is essential. Studies have revealed issues of contamination and the presence of unintended bacterial strains in commercial biofertilizers such as Herrmann and Lesueur (2013) performed the analysis on 65 commercial biofertilizers, and revealed that merely 37% of these products met the criteria for being labeled as “pure.” In contrast, a significant 63% of the tested biofertilizers exhibited contamination by one or more bacterial strains. Furthermore, in 40% of the cases, the tested products lacked the specified strains entirely and were instead found to contain contaminants. A shortage of suitable carriers for these formulations, inadequate storage facilities to prevent contamination and the unpredictability of their effectiveness due to extreme weather conditions add to the list of constraints. Additionally, the credibility of biofertilizer application can be undermined by the absence of crucial labeling information, such as expiration dates and the identification of microorganisms used in production. Most biofertilizers also exhibit selectivity in their actions, limiting their compatibility with certain chemical pesticides or fertilizers, which can affect integrated pest management or nutrient management programs. To overcome these challenges and limitations, continuous research, development, and collaboration among scientists, agricultural practitioners, and policymakers are imperative. It is crucial to explore and leverage the potential benefits of microbial formulations while actively addressing their drawbacks to advance sustainable agricultural practices.

## 10 Conclusion and future prospects

The primary focus in advancing agricultural productivity to meet the needs of our growing global population lies in investing in the development of microbial formulations. This greener approach supports plant growth and environmental sustainability. While bacterial strains often perform well in laboratory settings, their efficacy in field conditions is hindered by factors such as poor survivability, inappropriate carrier selection, or ineffective delivery methods. To ensure the success of bioformulations, the process begins with the critical task of selecting microbial strains carefully. These chosen strains must possess a competitive edge against native microflora while demonstrating beneficial functions even under stressful conditions, all the while maintaining their bio-efficacy once released. Creating an effective bioformulation demands several essential steps, including proper isolation and characterization of the microbial strains for their plant growth-promoting traits. Additionally, rigorous testing for pathogenicity is necessary to ensure bio-safety. Moreover, the selection of an ideal carrier is crucial to enhance the shelf life of the bioformulation and preserve its efficacy. Field conditions play a vital role in determining the success of a bioformulation. Therefore, it is imperative to assess the survival of the formulated product in real-world agricultural settings. The overall cost of developing and implementing the formulated product should be considered to ensure its feasibility and practicality on a larger scale.

Shifting the research focus towards the development of broad temperature and elevation ranged bioinoculants based bioformulation, harnessing their potential metabolites, holds the key to advancing sustainable and safe practices. Rather than solely concentrating on the isolation and characterization of new bacterial bioformulation, this approach offers several benefits by utilizing bioinoculants bioformulation that relies on potential metabolites, we can significantly enhance field efficacy while simultaneously addressing biosafety concerns. These bioformulations can be tailored to deliver targeted benefits, promoting plant growth, disease resistance, and nutrient uptake without the risk associated with introducing entirely new bacteria into the environment. Moreover, there is a pressing need to explore ways to stabilize these bioformulations and increase their shelf life. By doing so, we ensure their long-term viability and practicality for widespread agricultural adoption, promoting cost-effectiveness and convenience. To achieve this, research efforts should be directed toward identifying numerous inexpensive and non-toxic carrier materials. These materials can play a crucial role in preserving the bioformulations’ effectiveness and longevity, allowing farmers easy access to sustainable solutions without imposing harmful consequences on the environment or human health. Lastly, to truly replace agricultural chemicals and make agriculture more sustainable and productive, it is essential to investigate effective delivery methods. Implementing innovative delivery techniques can ensure that bioinoculant bioformulation reaches their target areas efficiently, maximizing their beneficial impact on crops and reducing the need for conventional chemical interventions. By emphasizing these research areas—developing specific bioinoculants bioformulation based on potential metabolites, stabilizing formulations, exploring eco-friendly carrier materials, and optimizing delivery methods—we pave the way for a more sustainable, productive, and environmentally friendly approach to agriculture.

## Author contributions

AK: Conceptualization, Writing – review & editing, Writing – original draft. AVS: Conceptualization, Supervision, Writing – review & editing. SG: Writing – original draft. AA: Writing – original draft. AP: Resources, Writing – original draft. VU: Writing – review & editing. BK: Writing – review & editing. VB: Writing – review & editing. AJ: Writing – review & editing. RG: Writing – review & editing.
